# Valorization of Lactate Esters and Amides into Value-Added Biobased (Meth)acrylic Polymers

**DOI:** 10.1021/acs.biomac.4c00891

**Published:** 2024-09-11

**Authors:** Marc Palà, Gerard Lligadas, Adrian Moreno

**Affiliations:** † Universitat Rovira i Virgili, Departament de Química Analítica i Química Orgànica, Laboratory of Sustainable Polymers, Tarragona 43007, Spain

## Abstract

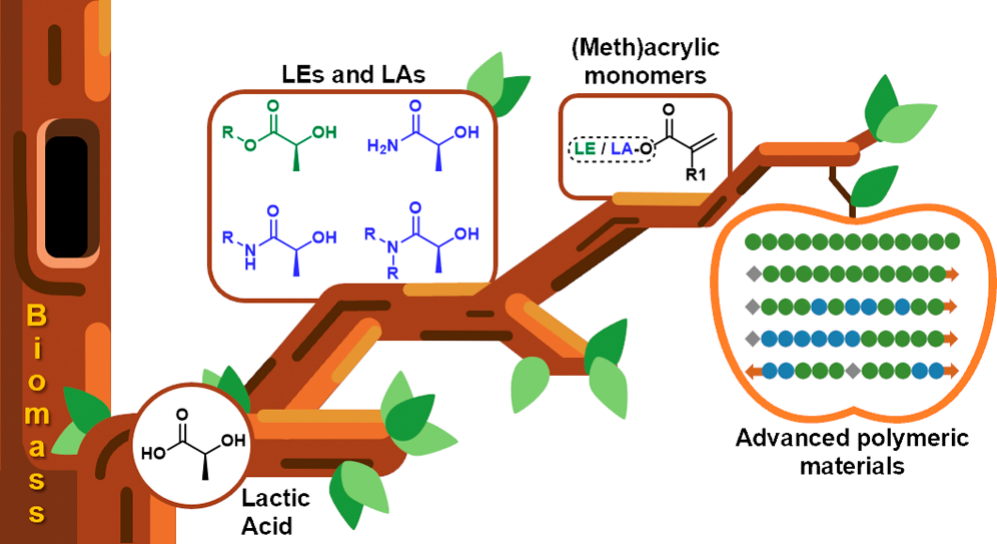

(Meth)acrylic polymers are massively produced due to their inherently attractive properties. However, the vast majority of these polymers are derived from fossil resources, which is not aligned with the tendency to reduce gas emissions. In this context, (meth)acrylic polymers derived from biomass (biobased polymers) are gaining momentum, as their application in different areas can not only stand the comparison but even surpass, in some cases, the performance of petroleum-derived ones. In this review, we highlight the design and synthesis of (meth)acrylic polymers derived from lactate esters (LEs) and lactate amides (LAs), both derived from lactic acid. While biobased polymers have been widely studied and reviewed, the poly(meth)acrylates with pendant LE and LA moieties evolved slowly until recently when significant achievements have been made. Hence, constraints and opportunities arising from previous research in this area are presented, focusing on the synthesis of well-defined polymers for the preparation of advanced materials.

## 1 Introduction

Traditional polymers derived from fossil fuels have become indispensable in contemporary human existence due to their exceptional chemical and physical attributes, endowing them with versatile properties such as lightweightness, durability, resilience, and resistance to decay.^1,2^ They have effectively supplanted numerous materials and substances, finding extensive utility across industrial, domestic, and medical domains as disposable implements, packaging materials, furnishings, machinery components, among others.^3−5^ Global production of polymers soared to 403 million tons in 2022 with forecasts predicting further escalation.^6^ However, the proliferation of polymers manufacture has elicited environmental apprehensions owing to their nonbiodegradable nature, persistent presence, and fossil-sourced origin.

In response to the challenges and environmental concerns associated with petroleum-based polymers, coupled with the shift toward the development of bioeconomies, extensive efforts have been made to upgrade plant biomass, commonly referred to as lignocellulosic biomass—comprising cellulose, hemicellulose, and lignin—toward advanced engineered polymeric materials.^7−10^ This is primarily due to its inedibility, cost-effectiveness, renewability, carbon neutrality, and widespread availability. For example, various processes such as pyrolysis, oxidation, hydrogenation, gasification, and microbial conversion have been employed for the depolymerization of lignin, yielding valuable low molecular weight compounds including phenol, vanillin, guaiacol, *p*-cresol, and catechols.^11,12^ Many of these compounds serve as crucial starting materials for the synthesis of innovative biobased polymers using different polymerization methods for diverse applications.^13,14^ Similarly, the isolation of sugar-rich fractions from cellulose and hemicellulose, followed by depolymerization, offers access to monomeric C5/C6 sugars, which serve as a platform for the production of valuable biobased chemicals such as furfural, levulinic acid, sorbitol, or xylitol, among others.^15−17^

**Figure 1:**
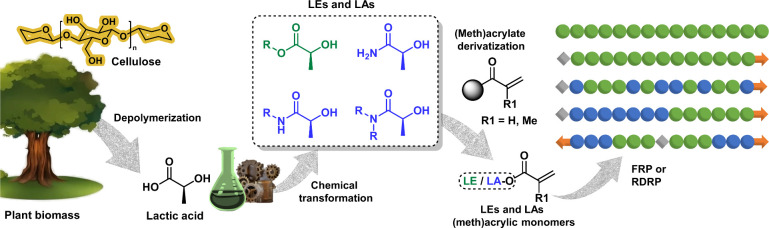
General overview of the topic that will be discussed in this review: The valorization of lactic acid into lactate esters (LEs) and amides (LAs) is used for the production of well-defined polymeric materials.

Among them, lactic acid has emerged as a crucial chemical platform globally. It serves as a fundamental material for synthesizing various chemicals, including acrylic acid, 1,2-propanediol, propylene glycol, oxalic acid, pyruvic acid, and 2,3-pentanedione, which have multiple applications in many industries.^18−20^ However, its predominant application lies in the production of polylactic acid (PLA), a biodegradable polymer that has garnered significant attention across diverse research domains, spanning from biomedicine to protective packaging, as extensively reviewed elsewhere.^21−27^ Less explored yet equally significant are lactate esters (LEs) and amides (LAs).^28^ These compounds have emerged as a green alternative to fossil-sourced solvents in industry and are used in many pharmaceutical and cosmetic applications due to their low toxicity. For instance, alkyl LEs are commonly used in food preservation,^29^ while alkyl LAs are employed in the agricultural sector as plant growth regulators and diluents for bactericides and fungicides.^30^ Among them, commercially available lactic acid derivatives such as ethyl lactate ester (EL) and *N*,*N*-dimethylactate amide (DML) stand in the portfolio of green biosolvents poised to substitute traditional chemicals due to their appealing blend of properties, including a high boiling point, minimal toxicity, and strong solvency. For instance, EL produced and commercialized under different trade names (Galaster EL 98.5 FCC, Galasolv 003, and VertecBio) by Galactic, Corbion, or Vertec Biosolvents is approved by the U.S. Food and Drug Administration (FDA) and extensively used as excipient in pharmaceutical compositions.^31^ On the other hand, DML is commercialized under the trade name Agnique AMD 3L by BASF and used in agrochemical formulations.^30^ Therefore, it is unsurprising that their utilization as solvents has seen a rise in various industrial sectors over recent years, encompassing specialty coatings,^32^ polymeric membrane preparation,^33−35^ organic reactions^36,37^ and polymerizations media,^38−42^ agrochemicals,^43^ and cytotoxicity assays.^44^ Several comprehensive reviews have been dedicated to the synthesis and applications of (meth)acrylic monomers and polymers derived from lignocellulosic biomass.^45−48^ Therefore, this concise review aims to shed light on the efforts undertaken regarding the synthesis and applications of (meth)acrylic monomers and polymers derived from LEs and LAs (Figure 1). The first reports on LEs and LAs-derived (meth)acrylic monomers and polymers date back to the 1940s. However, significant achievements have only been made in the last two decades, and particularly in the past 10 years, due to the application of reversible-deactivation radical polymerization (RDRP) techniques to their polymerization. These advancements have enabled access to well-defined polymers and the preparation of functional materials. We begin by identifying their key enabling synthetic methods and continue with an analysis of their transformation into free radical polymerization (FRP) polymers and RDRP well-defined homo-, random-, and block copolymers. Additionally, we also discuss the potential of these (meth)acrylic monomers for the development of advanced functional materials. Finally, we conclude with challenges and some perspectives on this exciting research area.

## 2 Synthesis of LE and LA (Meth)acrylic Monomers Derived from Biobased Lactic Acid

**Figure 2:**
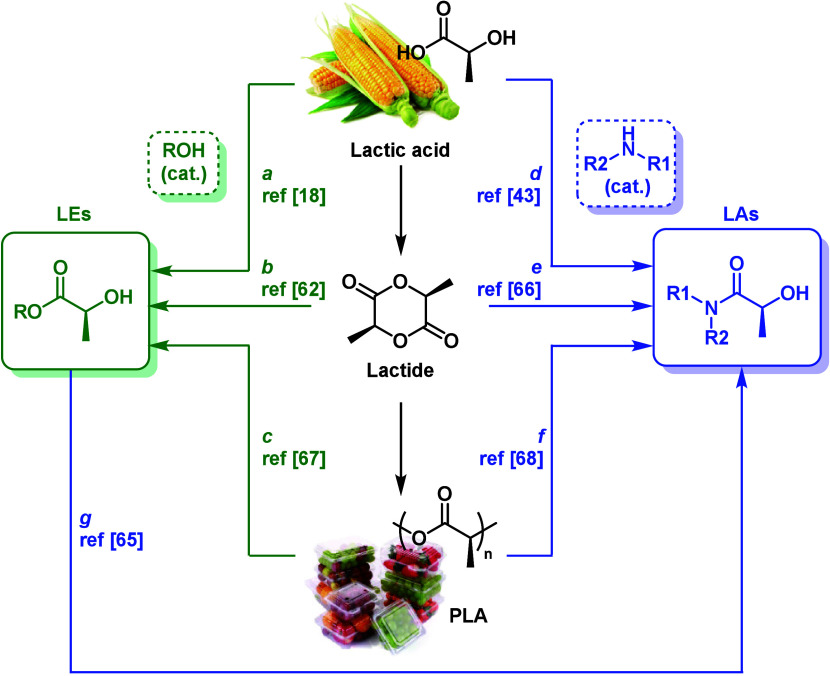
Chemical transformation routes of lactic acid and PLA into lactate esters (LEs) and lactate amides (LAs).

**Figure 3:**
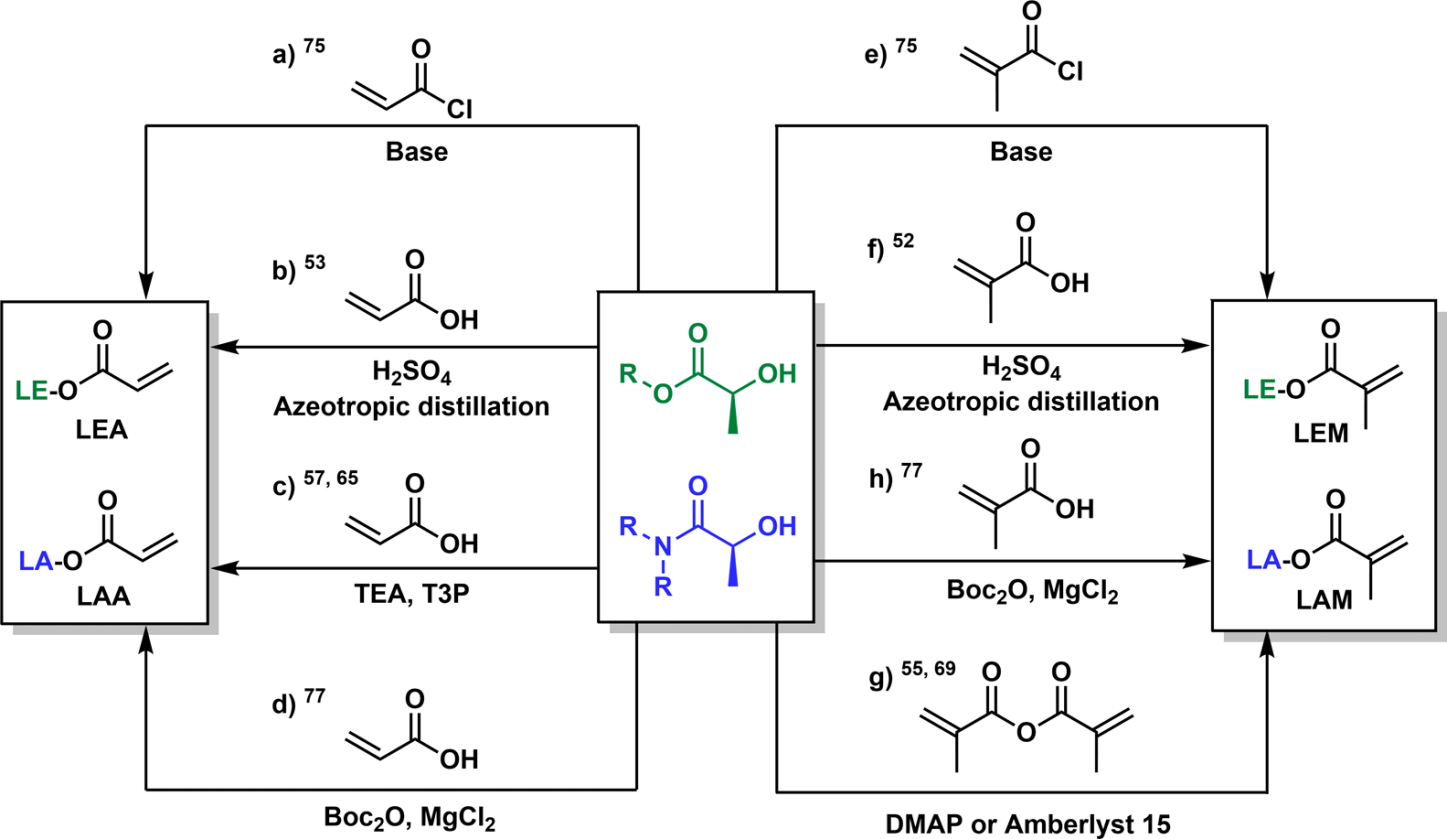
Synthetic routes for the preparation of methacrylate monomers derived from LEs and LAs.

Owing to the presence of a monohydroxyl functional group, LEs and LAs are prone to their transformation into meth(acrylate) monomers for radical polymerization aimed at the subsequent production of sustainable and advanced polymeric materials.^49−57^ The different routes developed so far for the preparation of these monomers involve the preparation of the corresponding LEs and LAs precursors (Figure 2), followed by the chemical derivatization of the secondary hydroxyl group to introduce the meth(acrylic) functionality (Figure 3). LEs are commonly produced through the catalytic esterification of lactic acid with linear alcohols such as methanol, ethanol, 2-propanol, isobutanol, *n*-butanol, and benzyl alcohol using homogeneous and heterogeneous catalysts such as acid ion-exchange resins (Figure 2, route a).^18,58−61^ However, they can also be synthesized from lactide—lactic acid dimer—through metal-catalyzed lactide alcoholysis (Figure 2, route b).^62^ In addition to lactic acid platform EL, it is also found naturally, albeit in small quantities, in various foods such as alcoholic beverages or fruits.^29^ This natural occurrence allows for its direct extraction through specific fermentation processes.^63,64^ Here, it is also important to mention that LEs such as EL are also important building blocks for the preparation of LAs via thermal-induced aminolysis, which can be accelerated using an organocatalyst (Figure 2, route g). For instance, tetrahydrofurfuryl amine was used for the preparation of the corresponding LA from EL in bulk using 1,5,7-triazabicyclo[4.4.0]dec-5-ene (TBD) as an organocatalyst.^65^ In this sense, LAs such as DML are also commonly prepared via lactic acid amidation or from lactide via ring-opening (Figure 2, routes d and e).^43,66^

Linked to use of LEs as building blocks for the preparation of LAs, PLAs have also emerged as an excellent platform for the preparation of LEs and LAs through the corresponding catalytic alcoholysis or aminolysis with the desired alcohol or amine (Figure 2, routes c and f).^67−70^ Although in the eyes of the public, PLA is often regarded as a “biodegradable” plastic, this statement is far from reality since the complete degradation of PLA in typical landfill conditions can take up to 100 years, and industrial composting requires controlled temperatures (60 °C) and constant feeding of microorganisms for degradation.^71^ Therefore, chemical upcycling routes, as mentioned above, appear to be an attractive and necessary strategy to reduce PLA accumulation and contamination, while producing valuable chemical synthons. For instance, *N*-lactoyl ethanolamine (*N*-LEA) was prepared through the aminolysis reaction from waste PLA filaments in the presence of ethanolamine for subsequent upcycling into 3D printable material.^68^

**Figure 4:**
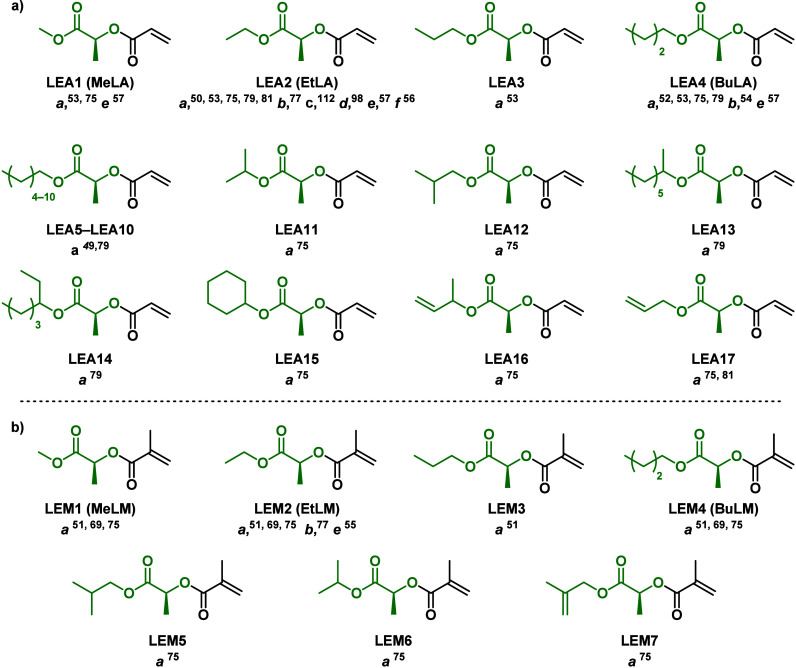
Library of (a) LEAs and (b) LEMs and the methodology employed for its polymerization. *a* = FRP, *b* = RAFT, *c* = PET-RAFT, *d* = RAFT-PISA, *e* = Cu(0)-wire SET-LRP, *f* = Cu(II)-mediated photopolymerization, and *g* = aqueous Cu(0)-mediated living radical polymerization.

Reaction with acryloyl chloride in the presence of triethylamine (TEA) using different solvents is the most common method to prepare acrylic monomers derived from LEs and LAs (Figure 3, route a).^72−74^ The LE acrylates (LEAs) and LA acrylates (LAAs) reported in the literature are gathered in Figure 4a and Figure 6a, respectively. Following this approach several alcohols, including methyl, ethyl, *n*-propyl, *n*-butyl, cyclohexyl, or allyl lactate, as well as DML, were transformed into the corresponding acrylate counterparts with high yields after purification by vacuum distillation.^55,56,75^ The acrylic group can also be introduced by esterification with potentially renewable acrylic acid, providing a greener alternative to the use of acryloyl chloride. A library of LE acrylates (LEAs) with linear alkyl groups from C1–C4 and C6–C12 was prepared via acid-catalyzed azeotropic distillation of the corresponding alcohol with acrylic acid in high yields (>82%) (Figure 3, route b).^49,50^ To avoid heating, which can cause partial oligomerization, the use of propylphosphonic anhydride (T3P) as an environmentally friendly ester coupler has also been demonstrated for the synthesis of ethyl lactate acrylate (EtLA). The same methodology is also valid for LAAs such as *N*,*N*-dimethyl lactamide acrylate (DMLA), and *N*-tetrahydrofurfuryl lactamide acrylate (THFLA), affording moderate yields (>60%) (Figure 3, route c).^56,65^ However, the relatively high cost of T3P can be a challenge when scaling up the reaction.^76^ Methacrylic monomers derived from LEs and LAs (LEMs and LAMs) are also accessible through reactions with the corresponding acyl chloride (Figure 3, route e). The LEMs and LAMs reported in the literature are gathered in Figure 4b and Figure 6b, respectively. Methyl, isobutyl, and *n*-butyl LEs were transformed into methacrylic monomers as reported by Rehberg and colleagues.^75^ Azeotropic distillation with methacrylic acid in the presence of methyl, ethyl, *n*-propyl, and *n*-butyl alcohols has also been used to obtain the corresponding LEMs (Figure 3, route f).^50,51^ Methacrylic acid was also used in an alternative and efficient route using di-*tert*-butyl dicarbonate (Boc_2_O) and catalytic amounts of MgCl_2_, yielding ethyl lactate methacrylate (EtLM) (Figure 3, route h).^77^ This innovative method allowed for subsequent polymerization, avoiding monomer purification in a one-pot strategy. Alternatively, methacrylic anhydride has also been used as a precursor following the Steglich esterification,^78^ with 4-(*N*,*N*-dimethylamino)pyridine (DMAP) for the preparation of EtLM and *N*-lactoyl ethanolamine dimethyl acrylate (DME), a difunctional methacrylate monomer derived from *N*-LEA (Figure 3, route g).^55,68^ Ultimately, a greener alternative has also been recently reported. This approach involves the use of an ion-exchange resin, commercially known as Amberlyst 15, as a potential alternative to DMAP in a heterogeneous catalyzed solvent-free reaction with methacrylic anhydride.^69^ Methyl lactate methacrylate (MeLM), EtLM, and *n*-butyl lactate methacrylate (BuLM) were obtained with high yield (>77%) (Figure 3, route g). In addition, Amberlyst 15 could be reused up to 10 times with no decrease in reaction conversion, highlighting the robustness of this approach and the potential of catalyst immobilization in heterogeneous structures.

## 3 FRP of LE and LA (Meth)acrylic Monomers

Polymerization of (meth)acrylic monomers through free radical polymerization (FRP) is a simple and straightforward method intensively used in both industry and academia for the preparation of polymeric materials. Lactate ester acrylates (LEAs) and methacrylates (LEMs), shown in Figure 4, were first polymerized by FRP as reported in 1945.^75^ However, this pioneering study was limited in exploring the reactivity of LEAs and LEMs in the radical polymerization process, neglecting the evaluation of physical properties of the obtained polymers. It was not until 2013 that the potential of LEAs was demonstrated in a patent publication.^79^ The UV-induced FRP of acrylic acid with LEAs containing a variable alkyl chain length such as *n*-ethyl (EtLA) and *n*-butyl (BuLA) alkyl moieties was shown to be an interesting approach to preparing pressure-sensitive adhesives with improved degradability under basic conditions due to the cleavage of pendant lactate ester chains. For instance, adhesive formulations containing BuLA showed a remarkable peel strength of 82.6 N·dm^–1^ and a time for adhesive debonding of about 10 min, together with a 76% weight loss after being washed with an aqueous alkali solution. Later on, LEAs and LEMs containing a variable alkyl chain (LEA1–4 in Figure 4) were also subject to FRP and demonstrated their potential as hydrogels due to their higher hydrophilicity, measured by moisture uptake, compared to poly(methyl methacrylate) (PMMA) and poly(methyl acrylate) (PMA).^49−53^ Reported glass transition temperatures for poly(LEAs) (PLEAs) containing linear C1 to C4 side groups decreased from −18.6 to −66.3 °C upon increasing the length of the alkyl lactate segment, while for their methacrylic counterparts (LEM1–4 in Figure 4), *T*_g_ varied from 119.5 to 72.1 °C.^51,53^

**Figure 5:**
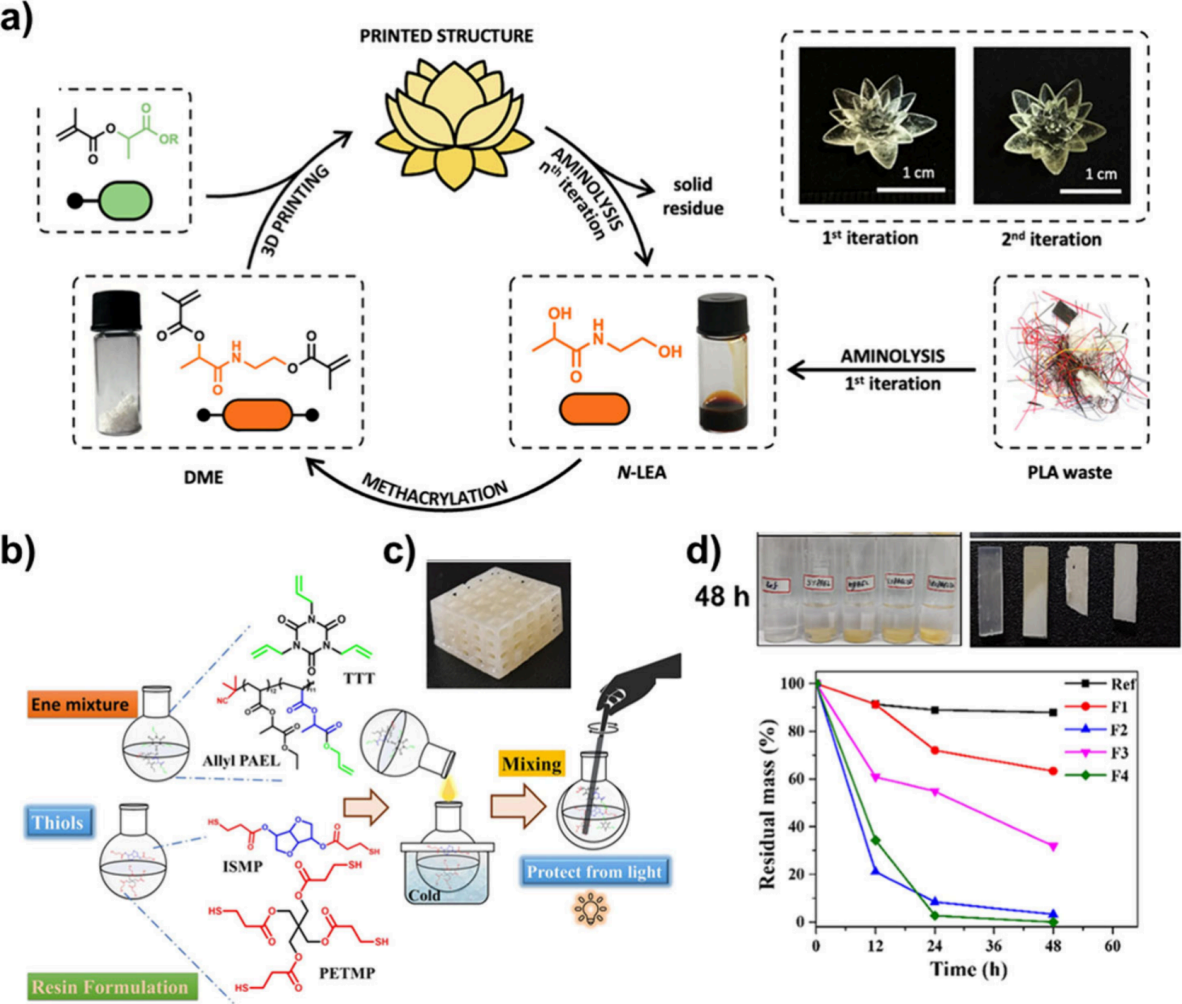
Use of LEAs and LEMs in digital light processing (DLP) 3D printing. a) Illustration of the printing process using LEMs and recycling process via aminolysis. Adapted from ref (69). Copyright 2024 American Chemical Society. b) Schematic representation of resin formulation using LEAs for the preparation of thiol–ene thermoset polymeric network. c) Highly porous complex CAD file 3D printed structure showing the fidelity of thiol–ene resin printability. d) Real-time photographs of degradation of the different 3D printed parts, and degradation plot after their exposure to 10 mL 1 M NaOH solution over 48 h. Adapted from ref (81). Copyright 2024 Elsevier.

**Figure 6:**
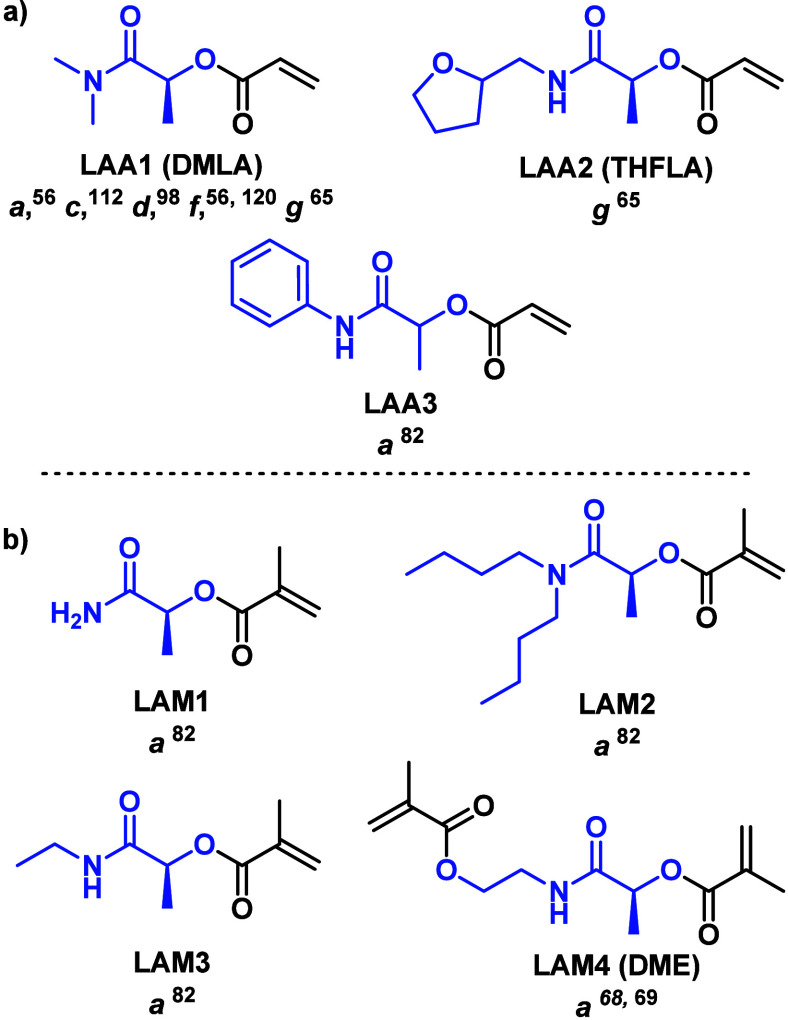
Library of (a) LAAs and (b) LAMs and the methodology employed for its polymerization. *a* = FRP, *b* = RAFT, *c* = PET-RAFT, *d* = RAFT-PISA, *e* = Cu(0)-wire SET-LRP, *f* = Cu(II)-mediated photopolymerization, and *g* = aqueous Cu(0)-mediated living radical polymerization.

More recently, FRP of LEAs and LEMs is also gaining momentum in new fabrication techniques such as additive manufacturing, also known as 3D printing. For instance, Blasco et al. recently reported the use of LEMs (methyl, ethyl, and butyl) as biobased monomers for digital light processing (DLP) 3D printing (Figure 5a).^69^ An interesting aspect of this work was the preparation and use of DME as a cross-linker (Figure 6), which was obtained by the chemical upcycling of PLA waste filaments via aminolysis using ethanolamine. This demonstrated the potential of lactic acid as a platform for the preparation of sustainable cross-linkers. Initial studies using an ink formulation with a ratio of 9:1 LE monomer to cross-linker and 2 wt % of photoinitiator were found to be printable, albeit that some overpolymerization events were appreciable. These issues were overcome by adding methacrylic acid (10 wt %) as a reactive diluent. Regardless of the LEM used in the ink formulation, complex 3D models such as a water lily were printable, resulting in very well-defined structures. Additionally, the storage modulus in the glassy state was found to decrease as the length of the alkyl chain of the ester increased (1.78 GPa for MeLM, 1.62 GPa for EtLM, and 1.08 GPa for BuLM), owing to the plasticizer effect of the alkyl chains. These results were comparable to other biobased thermosetting resins based on vanillin- and eugenol-based monomers,^80^ and they suggest the possibility of modifying the cross-linking degree of the resin formulations (with a mixture of LEAs) to prepare multimaterial models featuring both soft and rigid domains. Ultimately, a key aspect of this work was the possibility of chemically upcycling the printed structures via aminolysis. The authors demonstrated that conducting aminolysis with ethanolamine allowed the complete dissociation of the thermoset printed structure, yielding *N*-LEA, the precursor of the initial cross-linker. Once the upcycling was performed, methacrylation of the cross-linker precursor allowed the preparation of the cross-linker and made it possible to close the loop by preparing new printed samples (Figure 5a).

In a similar domain, Pal et al. recently demonstrated that the preparation of degradable thiol–ene polymeric networks based on poly(ethyl lactate acrylate) (PEtLA) is possible using digital light processing (DLP) 3D printing.^81^ In this study, the authors conducted partial hydrolysis (50%) of PEtLA under basic conditions (LiOH) to form carboxylic acid groups. These groups were then reacted with allyl bromide to install allyl groups, enabling the preparation of thermoset resins via a thiol–ene click reaction. Resin formulations consisted of modified PEtLA and triallyl-1,3,5-triazine-2,4,6(1H,3H,5H)-trione (TTT) as allyl-containing compounds, while difunctional thiol derived from isosorbide (ISMP) and multifunctional pentaerythritol tetrakis(3-mercaptopropionate) (PETMP) were used as cross-linkers (Figure 5b). These formulations were found to be printable via thiol–ene photoinduced polymerization, producing high-quality specimens such as 3D-printed teeth models and complex porous materials used for calibrating DLP 3D printers, thereby validating the capabilities of the aforementioned thiol–ene resins (Figure 5c). It is noteworthy that the introduction of allyl-modified PEtLA improved the mechanical properties of the resins compared with previously reported resins, which was attributed to the higher cross-linking density formed during the 3D printing process. Importantly, the hydrolytic degradation of the 3D-printed cross-linked thiol–ene network polymers was investigated under both acidic and basic conditions. While no degradation was observed under acidic conditions, near to quantitative degradation (95%) occurred within 48 h under basic conditions for thermosets containing 10% allyl-modified PEtLA (Figure 5d, F2 and F4), compared to only 10% degradation for a commercial thermoset material (Figure 5d, ref). Formulations containing 5% allyl-modified PEtLA experienced 60% degradation after 48 h, highlighting the importance of the amount of PEtLA in achieving complete degradation (Figure 5d, F3). This rapid degradation efficiency of 3D-printed parts within a span of 2 days corresponds to the lability of the ester linkages present in the ethyl lactate ester under basic conditions. These results demonstrate that these materials are appealing for various prototype applications, where a sustainable end-of-life is a requirement.

Similarly to LEAs and LEMs, LAAs and LAMs were also polymerized for the first time in 1947 using FRP in an old patent application.^82^ A modest scope of LAAs and LAMs, as shown in Figure 6, were subjected to FRP, affording polymers with variable solubility in organic solvents. It is worth mentioning that LAAs and LAMs were also copolymerized with (meth)acrylic acid, vinyl acetate, or styrene, giving rise to copolymers with different physical appearances. For instance, the copolymer of LAA3 with styrene afforded a soft rubbery material, while the copolymer of LAM1 with methacrylic acid led to a hard copolymeric resin. These early results demonstrated that the combination with conventional vinyl monomers is possible, thus envisioning their potential in the development of functional materials. However, the evaluation of the physical properties was limited to visual observations, and the solubility in relevant solvents such as water was not explored, thus masking the potential of LAAs and LAMs until they were recently applied in RDRP processes (*vide infra*).

## 4 Well-Defined RDRP Homopolymers from LE and LA-Derived Meth(acrylates)

Albeit FRP is effective for the preparation of polymeric materials, advanced applications often require careful control over the polymeric architecture. In this context, advanced polymerization techniques are indispensable for the preparation of well-defined polymeric architectures.^83^ Reversible-deactivation radical polymerization (RDRP) methods allow high control over targeting the desired molecular weight while maintaining low dispersity (*Đ*) and a high level of end group functionality. This is achieved through the fast and reversible activation of dormant polymer species, reducing the number of active radicals in the reaction medium and thus lowering the probability of termination by recombination or disproportionation. Consequently, polymer chains grow linearly over time up to the “programmed” degree of polymerization, preserving a narrow molecular weight distribution and most of the chain end groups, which are crucial for accessing more sophisticated macromolecular structures. For these reasons, RDRP methods play a crucial role in the development of high-value-added materials through well-defined polymer architectures such as homopolymers, copolymers, graft, or star-like polymers.^84−86^ The most relevant techniques include nitroxide-mediated polymerization (NMP),^87^ reversible addition–fragmentation chain transfer polymerization (RAFT),^88^ and a variety of Cu-mediated radical polymerization methods.^89−91^ For more information about RDRP methods, we direct readers to excellent and recent reviews.^86,92−94^

**Figure 7:**
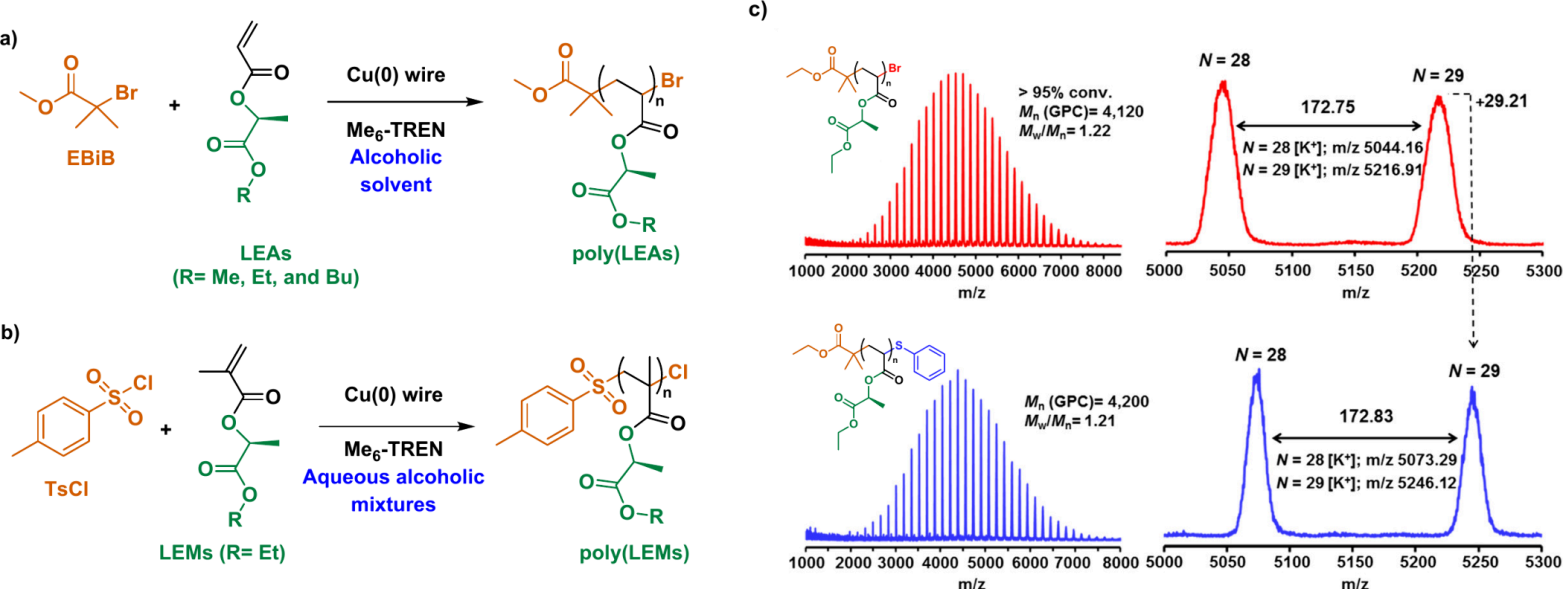
Well-defined homopolymers from LEAs and LEMs were analyzed using SET-LRP. a) Cu(0) wire/Me_6_-TREN-catalyzed SET-LRP of a) LEAs initiated with EBiB in alcoholic solvents and b) LEMs initiated with *p*-toluenesulfonyl chloride (Ts-Cl) in aqueous alcoholic mixtures. c) MALDI-TOF spectra of PEtLA obtained at 97% conversion before and after thio-bromo “click” modification with thiophenol. Magnified regions confirm the expected peak-to-peak spacing for the EtLA repeating unit and the near-perfect bromine chain-end functionality of the synthesized polymer. Adapted from ref (57). Copyright 2019 American Chemical Society.

The first example of RDRP of LEAs was reported in our laboratory in 2018.^57^ Our initial studies focused on copper-mediated RDRP (Cu-RDRP) techniques as is the case of single electron transfer-living radical polymerization (SET-LRP). The initial rational choice of SET-LRP was based on the operational simplicity of the technique, the use of robust and stable Cu(0) wire as a catalyst, and its excellent compatibility for polymerizing a wide variety of vinylic monomers at room temperature.^93^ In this sense, well-defined homopolymers from MeLA, EtLA, and BuLA were obtained using activated Cu(0)-wire, tris[2-(dimethylamino)ethyl]amine (Me_6_TREN), and ethyl α-bromoisobutyrate (EBiB) as the catalyst, ligand, and monofunctional initiator, respectively (Figure 7a). Aiming for a sustainable process, low carbon alcohols, ethyl lactate, and ethyl lactate–water mixtures were used to deliver PLEAs in less than 4 h at 25 °C, reaching high conversion (>90%) for molecular weights up to 65,000 g·mol^–1^, and narrow dispersity (*Đ* < 1.26). Later on, we also applied the same technique to successfully achieve the polymerization of EtLM in binary mixtures of organic solvent and water.^55^ As in the case of the acrylate counterpart, high conversion (85%) was reached using *p*-toluenesulfonyl chloride (TsCl) as the initiator after 4 h at 50 °C affording a polymer of molecular mass of 30,000 g·mol^–1^ and low dispersity (*Đ* = 1.2) (Figure 7b). Regardless, the monomer nature (acrylate or methacrylate), the high retention of chain-end functionality was confirmed through MALDI-TOF analysis. For instance, the MALDI-TOF spectrum of PEtLA, isolated at high conversion rates (>95%) both before and after end-group modification via thiol-bromo “click” reaction,^95^ demonstrated minimal side reactions and high bromine chain end-group fidelity after SET-LRP (Figure 7c).

**Figure 8:**
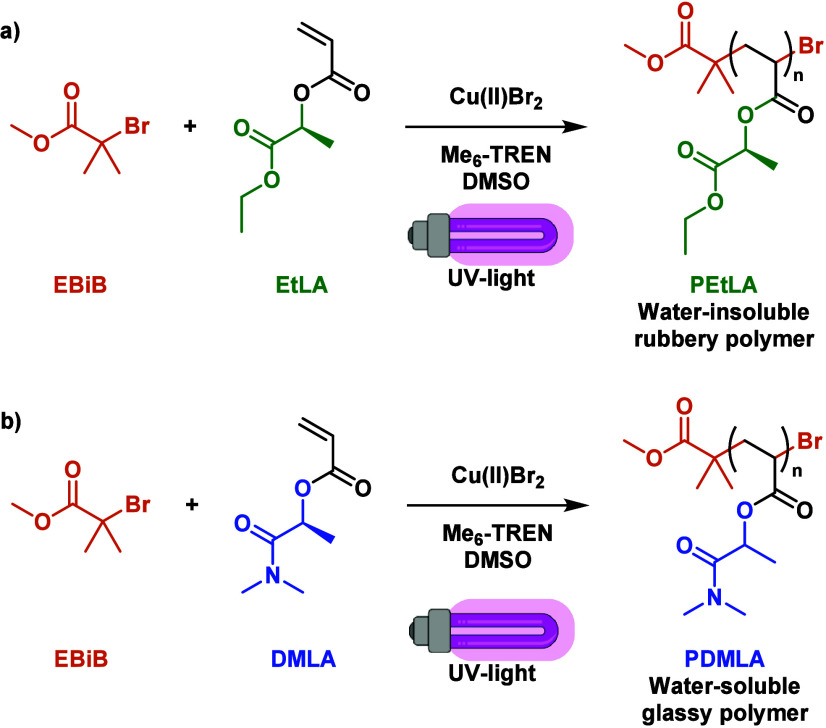
Cu(II)-catalyzed photoinduced radical polymerization of a) EtLA and b) DMLA.

In addition to that, our group also reported the preparation of homopolymers from EtLA and DMLA via Cu(II)-catalyzed photoinduced radical polymerization (Figure 8a,b).^56^ This alternative Cu-RDRP methodology offers the possibility to use light (ON/OFF) to switch the polymerization process and control the growth of the polymer chains over time, while also using minimal amounts of copper to catalyze the overall process.^96^ Controlled polymerizations took place after UV irradiation (λ_max_ ≈ 365 nm) a mixture containing Cu(II)Br_2_ and Me_6_TREN in DMSO to afford EtLA and DMLA homopolymers with high (>94%) conversions, molecular weights up to 75,000 g·mol^–1^, and *Đ* < 1.26. Poly(*N*,*N*-dimethyl lactamide acrylate) (PDMLA) exhibited remarkably higher *T*_g_ values (≈ 66 °C) in contrast to PEtLA (≈ −10 °C), probably due to stronger intermolecular attractive interactions from the polar *N*,*N*-dimethyl amide group. Also interesting, while PEtLA was found insoluble in water, PDMLA was found to be fully water-soluble, thus confirming their potential for the development of complete lactic acid-derived amphiphilic copolymers (*vide infra*).

**Figure 9:**
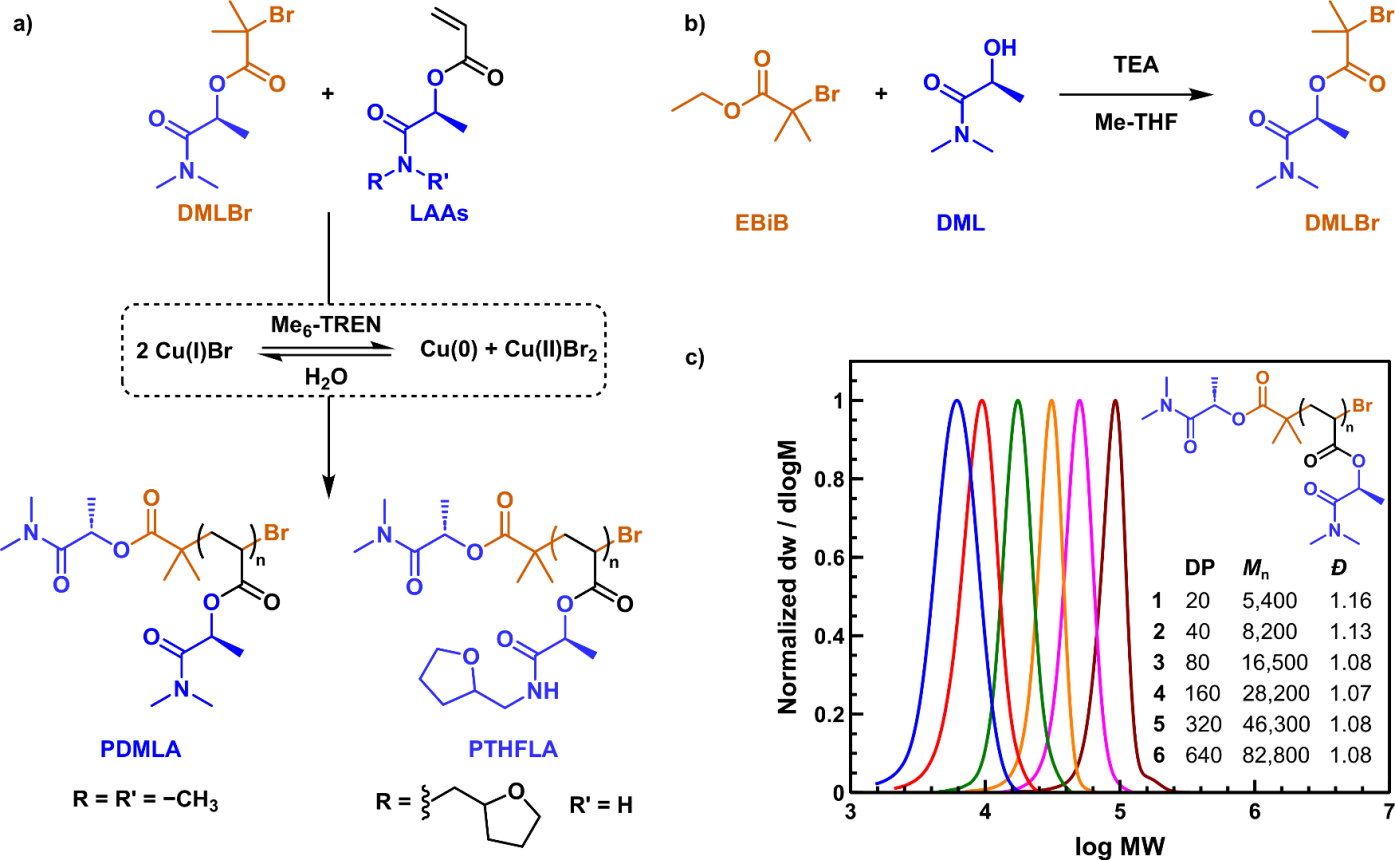
a) Aqueous Cu(0)-mediated living radical polymerization of DMLA and THFLA. b) Synthesis of the water-soluble initiator DMLBr from DML solvent. c) SEC traces (normalized to the peak height) of PDMLA prepared via aqueous Cu(0)-mediated living radical polymerization, targeting various degrees of polymerization (DPs) ranging from 20 to 640. Adapted from ref (65). Available under a CC-BY 3.0 DEED license. Copyright 2022 Royal Society of Chemistry.

Owing the water solubility of DMLA, we also explored the precise synthesis of DMLA and THFLA homopolymers via aqueous Cu(0)-mediated living radical polymerization (Figure 9a).^65^ This methodology takes advantage of the immediate—minutes—full disproportionation of CuBr/Me_6_TREN complex in water to highly active Cu(0) powder and CuBr_2_, which allows ultrafast—typically less than 1 h— polymerization of water-soluble vinyl monomers.^97^ Fast polymerization (<1 h) in water at 0 °C was achieved by self-regulated control by the *in situ* generated Cu(0) and Cu(II)Br_2_, yielding polymers up to 83,000 g·mol^–1^ with narrow dispersity (1.05 < *Đ* < 1.16) (Figure 9c). Here it is also important to mention that DML was functionalized with a bromoisobutyryl group and employed as a water-soluble initiator (Figure 9b), thus highlighting the potential of DML not only for the preparation of acrylic monomers but also for demanding initiators for radical polymerization in aqueous solution. Interestingly, poly(*N*-tetrahydrofurfuryl lactamide acrylate) (PTHFLA) exhibited temperature responsiveness in water solution, which in combination with the high hydrophilicity of PDMLA was exploited to develop thermoresponsive random copolymers (*vide infra*).

**Figure 10:**
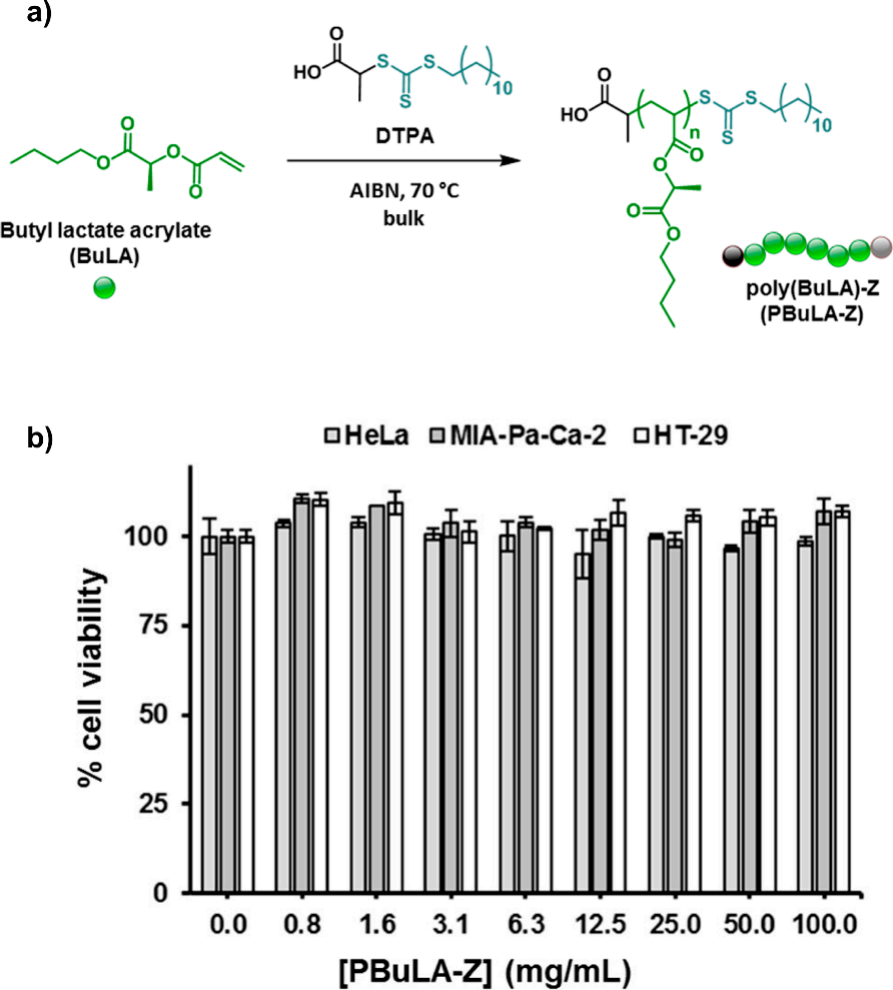
a) Reversible addition–fragmentation chain transfer (RAFT) polymerization using a DTPA as monofunctional chain transfer agent (CTA), to yield PBuLA homopolymers. b) CellTiter cell viability assay results for PBuLA at different concentrations assessed in different cell lines. Adapted from ref (54). Available under a CC-BY 3.0 DEED license. Copyright 2020 Multidisciplinary Digital Publishing Institute.

In order to explore the versatility of LEAs employing different polymerization techniques, our laboratory also reported the preparation of well-defined PLEAs through RAFT polymerization.^54^ Poly(butyl lactate acrylates) (PBuLA) homopolymers were prepared using 2-(dodecylthiocarbono-thioylthio)propionic acid (DTPA) as a RAFT agent and 2,2′-azobis(2-methylpropionitrile) (AIBN) as a polymerization initiator in bulk (Figure 10a). Size exclusion chromatography (SEC) analysis revealed a narrow MWD (1.22 < *Đ* < 1.27) for molecular weights up to 80,300 g·mol^–1^, reaching high monomer conversions (>90%) in 2 h. Cytotoxicity studies on various cell lines showed that exposure to PBuLA did not reduce cell viability (Figure 10b), indicating the potential application of (meth)acrylic polymers derived from LEs and LAs in the biomedical field (see Section 7). Later on, the polymerization of DMLA by RAFT aqueous polymerization was also reported to produce a hydrophilic PDMLA macromolecular chain transfer agent (macro-CTA) suitable for polymerization-induced self-assembly (PISA) (see Section 5),^98^ demonstrating the versatility of acrylic monomers derived from LEs and LAs to different polymerization techniques and reaction media.

## 5 Well-Defined Glassy and Rubbery Block Copolymers from LE and LA-Derived (Meth)acrylic monomers

**Figure 11:**
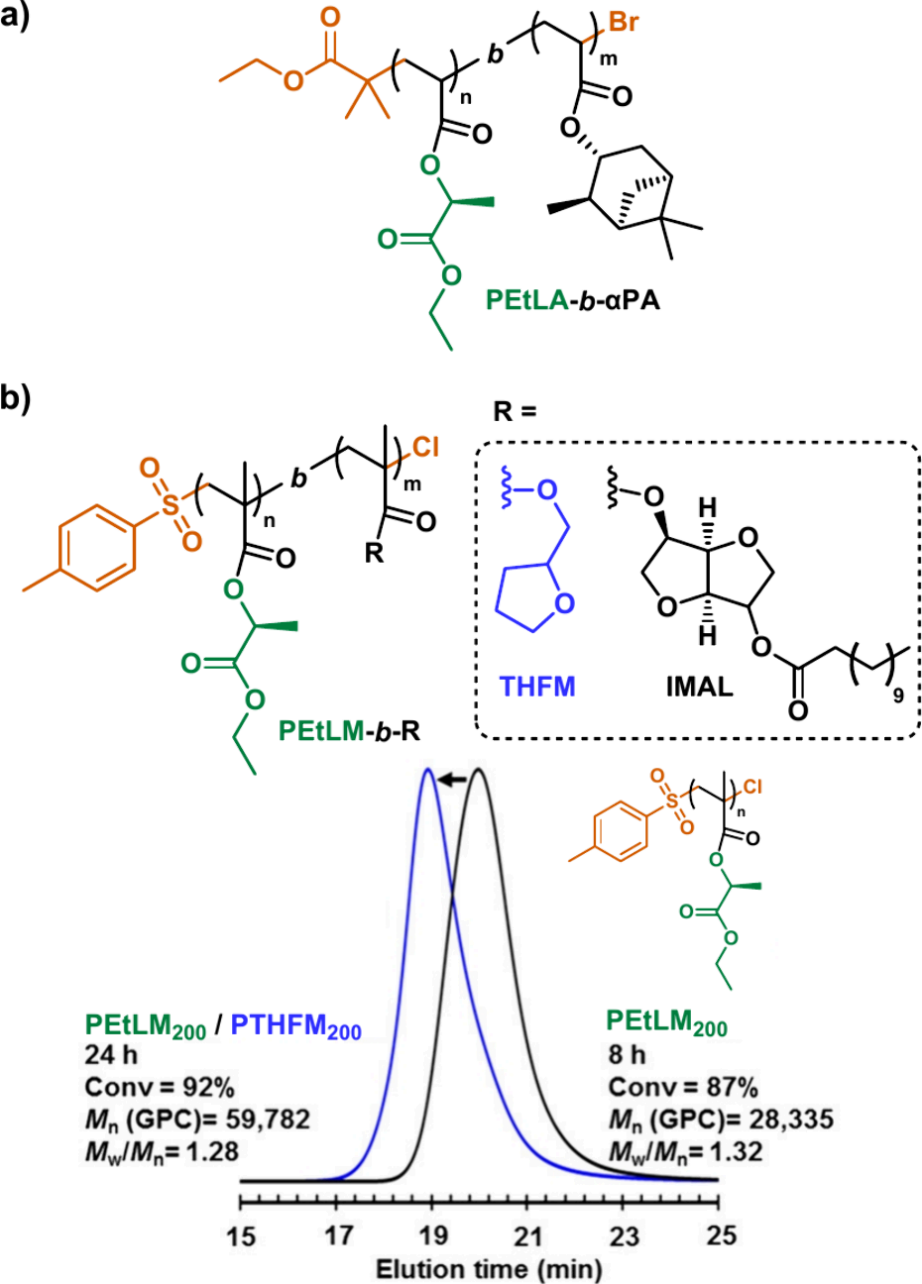
Well-defined block copolymers derived from a) LEAs and b) LEMs via *in situ* sequential chain extension. c) SEC traces of polymer before and after in situ block copolymerization of PEtLM with THFM. Adapted from ref (55). Copyright 2019 American Chemical Society.

As discussed in the previous section, RDRP allows for near-quantitative monomer conversion at various DPs and high chain end-group retention of homopolymers derived from LEAs/LEMs and LAAs/LAMs. In this sense, the combination of both factors also opens up the possibility of preparing block copolymers through the *in situ* sequential addition of a second monomer (i.e., *in situ* chain extension of the first block at near complete conversion). This approach was first successfully applied in our laboratory with in the preparation of AB-block copolymers (Figure 11). For instance, block copolymers from EtLA and α-pinene acrylate (αPA), a biobased monomer from turpentine,^99^ were prepared using SET-LRP and showed two distinctive *T*_g_ from PEtLA and poly(αPA) segments at 7 and 65 °C, respectively (Figure 11a).^57^ Similarly, phase separation was also observed in AB block copolymers (Figure 11b) containing EtLM and tetrahydrofurfuryl methacrylate (THFM), or EtLM and D-Isosorbide 2-laurate-5-methacrylate (IMAL)^55^ indicating immiscibility between blocks and envisioning their potential application for the preparation of ABA sustainable thermoplastic elastomers (TPEs). In all the above-discussed cases, the well-defined nature of block copolymers was assessed through SEC analysis, where a distinct shift of the SEC corresponding to the first monomer (EtLA or EtLM) toward higher molecular weight ranges, without significant shoulders or tailing was observed, indicating a successful incorporation of the second monomer segment (Figure 11c).

**Figure 12:**
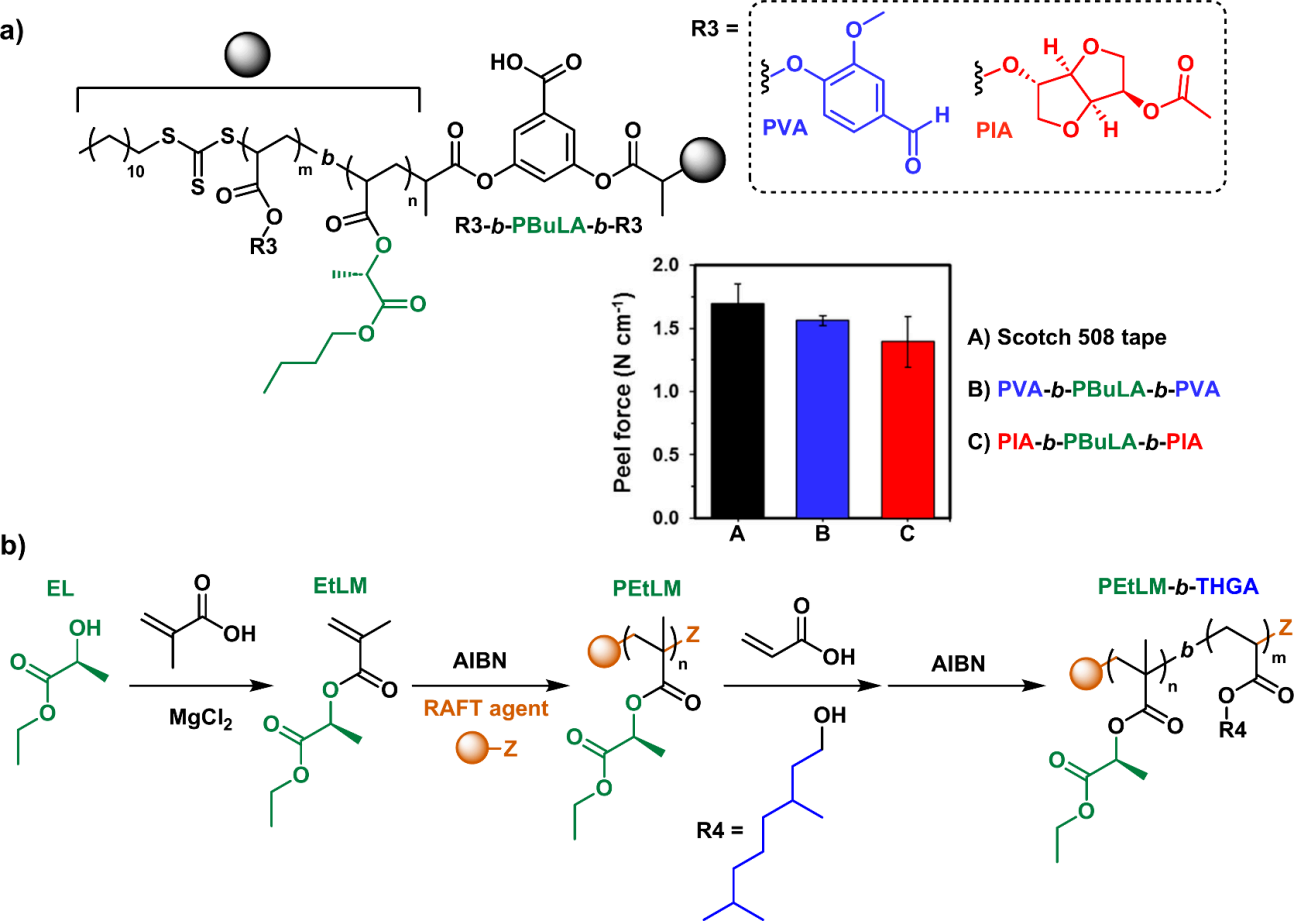
a) ABA block copolymer derived from LEAs and its application as pressure sensitive adhesives. b) One-pot synthetic strategy for the preparation of well-defined AB block copolymers derived from LEMs.

RAFT polymerization was also demonstrated as an efficient polymerization tool to prepare ABA block copolymers derived from LEAs. In this case, the difunctional RAFT agent, 3,5-bis(2-dodecylthiocarbonothioylthio-1-oxopropoxy)benzoic acid (BTCBA), was used for the synthesis of a PBuLA (*T*_g_ = −21 °C) macroinitiator in bulk as a soft segment.^54^ Subsequently, chain extension with poly(isosorbide 2-acrylate-5-acetate) (PIA, *T*_g_ ≈ 76 °C)^100^ or poly(vanillin acrylate) (PVA, *T*_g_ ≈ 80 °C)^101^ blocks as hard segments in a Rhodiasolv PolarClean green solvent yielded ABA-type TPEs, which were tested as a pressure sensitive adhesive (PSA) with 180° peel strength of about 1.5 N·cm^–1^, a result comparable to commercially available tapes with a peel strength of 1.7 N·cm^–1^ (Figure 12a). In a similar domain, poly(ethyl lactate methacrylate) (PEtLM) was also prepared by RAFT polymerization in one-pot synthesis from EL (Figure 12b), thus avoiding monomer isolation and purification,^77^ showcasing the robustness of the polymerization of these monomers in the presence of solvent impurity traces. Thermal analysis of PEtLM revealed a *T*_g_ around of 47 °C. In addition, chain extension was also possible after addition of desired alcohol in combination with acrylic acid. AB and ABA copolymers containing PEtLM as hard block and poly(tetrahydrogeraniol methacrylate) as central soft block (*T*_g_ = −27 °C) exhibited phase separation as stated by thermal analysis demonstrating the potentiality of this one-pot strategy for development of advanced materials (Figure 12b).

### 5.1 Well-Defined Block Polymers from DMLA and EtLA for Solution-Assembly Applications

**Figure 13:**
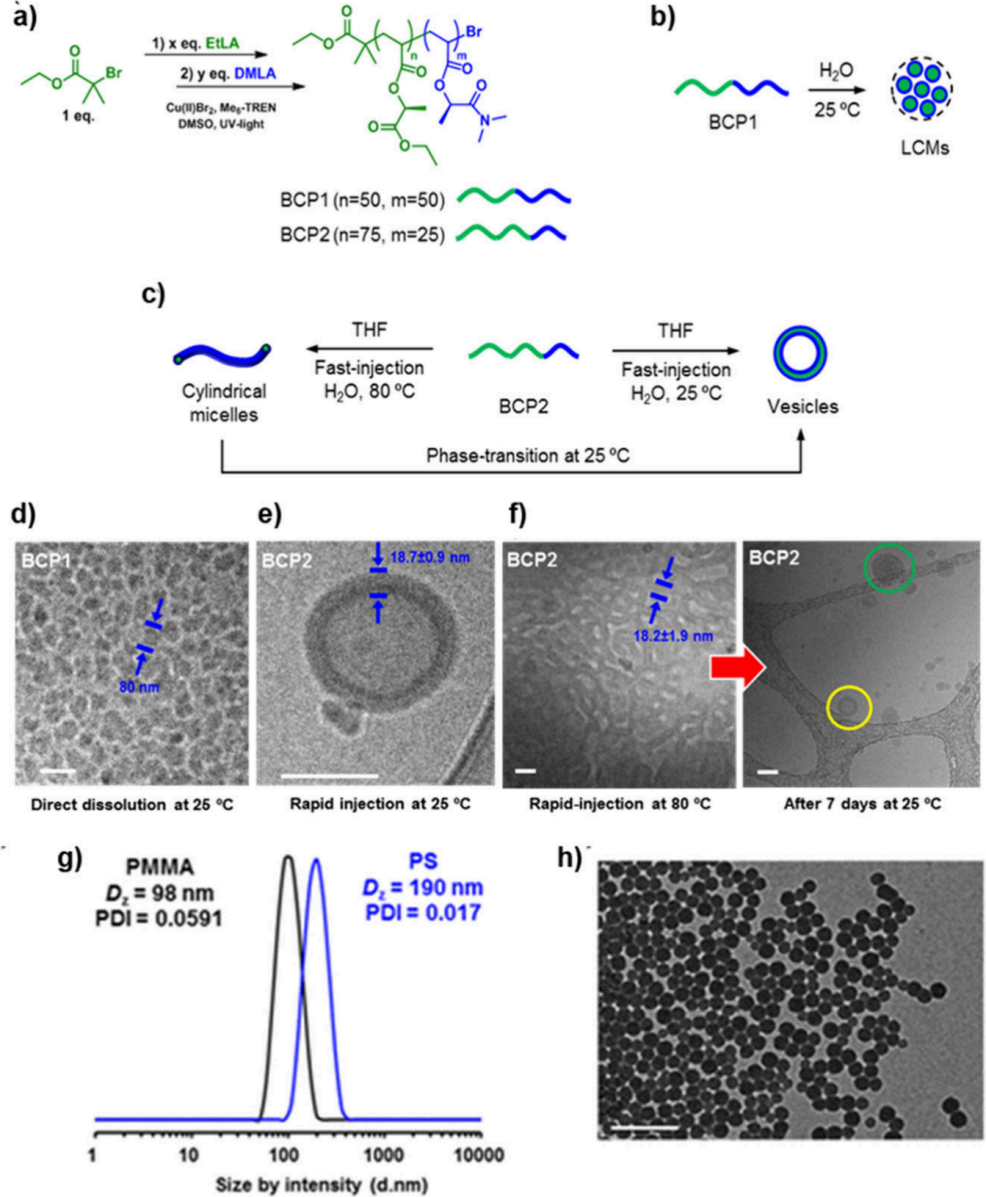
Synthesis of amphiphilic block copolymers derived from DMLA and EtLA and evaluation of their self-assembly behavior and potential application as surfactants. a) Synthesis of amphiphilic block copolymers from EtLA and DMLA via Cu(II)-mediated radical photopolymerization. b) BCP1 micelles self-assemble in water at 25 °C. c) BCP2 vesicles self-assemble when the copolymer is injected into water at 25 °C but BCP2 worm-like micelles self-assemble when the injection is carried out at 80 °C. The worm-like micelles transform into vesicles after some days at 25 °C. Cryo-transmission electron microscopy (cryo-TEM) images for d) BCP1 self-assembled in water and e) BCP2 self-assembled by fast injection of the copolymer (dissolved in THF) into water at 25 °C. f) Cryo-TEM images recorded 30 min after self-assembly of BCP2 by fast injection of the copolymer (dissolved in THF) into hot water (80 °C) (left image) and after 7 days at 25 °C (right spectrum). g) DLS size distribution by intensity of PMMA and PS latexes stabilized by BCP1. h) TEM image of the PMMA latex. Scale bar in all microscopy images is 100 nm. Adapted from ref (56). Copyright 2019 American Chemical Society.

Amphiphilic block copolymers (ABPCs) composed of both hydrophobic and hydrophilic segments represents an excellent platform to access advanced nanostructured morphologies such as micelles, vesicles and nanofibers among others.^102,103^ ABCPs find numerous applications in medical, pharmaceutical, cosmetic, and agricultural formulations as emulsifiers and dispersants, in cleaning and detergent products (as foamers, rinse aids, cosurfactants, and antiredeposition agents), in food processing (as emulsifiers and gelling agents), in the paper, paint, and coatings industry (as dispersants), and in polymer chemistry (as compatibilizers and in emulsion polymerization).^104−107^ Therefore, recent years have witnessed a cohesive interest in the development of biobased ABCPs that meet market requirements and offer the possibility of gradually substituting those derived from petroleum fossil resources. In this sense, our laboratory was the first envisioning that the different water solubility exhibited by the corresponding homopolymers—PEtLA, being hydrophobic and water-insoluble, and PDMLA, being hydrophilic and water-soluble—could present a unique opportunity to develop sustainable ABCPs tailored for specific applications.^56^ Thus, our initial experiments in this area involved the preparation of PEtLA/PDMLA copolymers via a one-pot polymerization approach (i.e., *in situ* chain extension of the first block with a second monomer) as already discussed in the previous section. The polymerizations were conducted through Cu(II)-mediated living radical photopolymerization. Briefly, after the hydrophobic core (PEtLA) was completely polymerized, DMLA was added to generate block copolymers without requiring intermediate purification steps. Two distinct PEtLA/PDMLA copolymers were synthesized, with hydrophobic/hydrophilic ratios set at 50/50 (BCP1) and 75/25 (BCP2) (Figure 13a). With the two PEtLA/PDMLA copolymers with different composition in-hand, their self-assembly in water was evaluated via direct dissolution in water or solvent-exchange methodology (i.e., addition of copolymer solution in organic solvent to a water solution or vice versa). In the case of BCP1, aggregation of individual spherical micelles into larger compound micelles of about 80 nm was observed through cryo-TEM analysis (Figure 13b,d). The observed morphology was attributed to the presence of attractive secondary interactions, such as hydrogen bonding and dipole–dipole interactions, which ultimately induce the hydrophobic swelling of micellar coronas and the formation of nonuniform micellar clusters. On the other hand, the self-assembly of BCP2, characterized by a higher hydrophobic nature, led to the formation of unilamellar vesicles with diameters around 50–150 nm and a membrane thickness of 18 nm, as observed by cryo-TEM (Figure 13c, right, and e). Additionally, a phase transition between lyotropic phases was noted during the preparation of BCP2 nanoassemblies at a temperature above the *T*_*g*_ of both monomers (80 °C) (refer to Figure 13c, left). Briefly, cryo-TEM analysis revealed the formation of worm-like micelles with a diameter of 18 nm, consistent with the membrane thickness of unilamellar vesicles prepared at room temperature, which subsequently evolved into unilamellar vesicles (highlighted by yellow circles) and onion-like vesicles (highlighted by green circles) after 7 days (Figure 13f). The initial assembly into worm-like micelles was attributed to a higher swelling of the hydrophilic block (PDMLA), resulting in a larger hydrophilic interfacial area compared to the cross-section of the hydrophobic block, shifting the packing parameter to the range of worm-like micelles.^108^ Overall, these results highlight the potential of PEtLA/PDMLA copolymers to develop different morphologies by simple modifications of block length and preparation method.

The potential applicability of PEtLA/PDMLA copolymers in industrially relevant processes was evaluated by employing them as macromolecular surfactants in emulsion polymerization. As a proof of concept, BCP1 was utilized to stabilize emulsion suspensions of industrially relevant monomers such as methyl methacrylate (MMA) and styrene (S) in water, facilitating their free radical polymerization without the presence of a coagulum process. Near-quantitative conversions were achieved for both monomers, resulting in stable latex dispersions. DLS and TEM analyses confirmed the presence of spherical latex dispersions with a narrow PDI for both monomers (Figure 13,g,h). In addition, latex dispersions were found stable when exposed to challenging conditions, such as extreme pH conditions (acidic: pH = 1, basic: pH = 14), low temperature (4 °C), or the presence of electrolytes (NaCl), which not only stand but also surpass the performance of nonbiobased and biobased surfactants that tends to coagulate at acidic or basic pH, as well as in the presence of saline concentrations where stabilization forces are no longer effective.^109,110^ Therefore, these results envision a high potential of PEtLA/PDMLA copolymers as effective surfactants.

**Figure 14:**
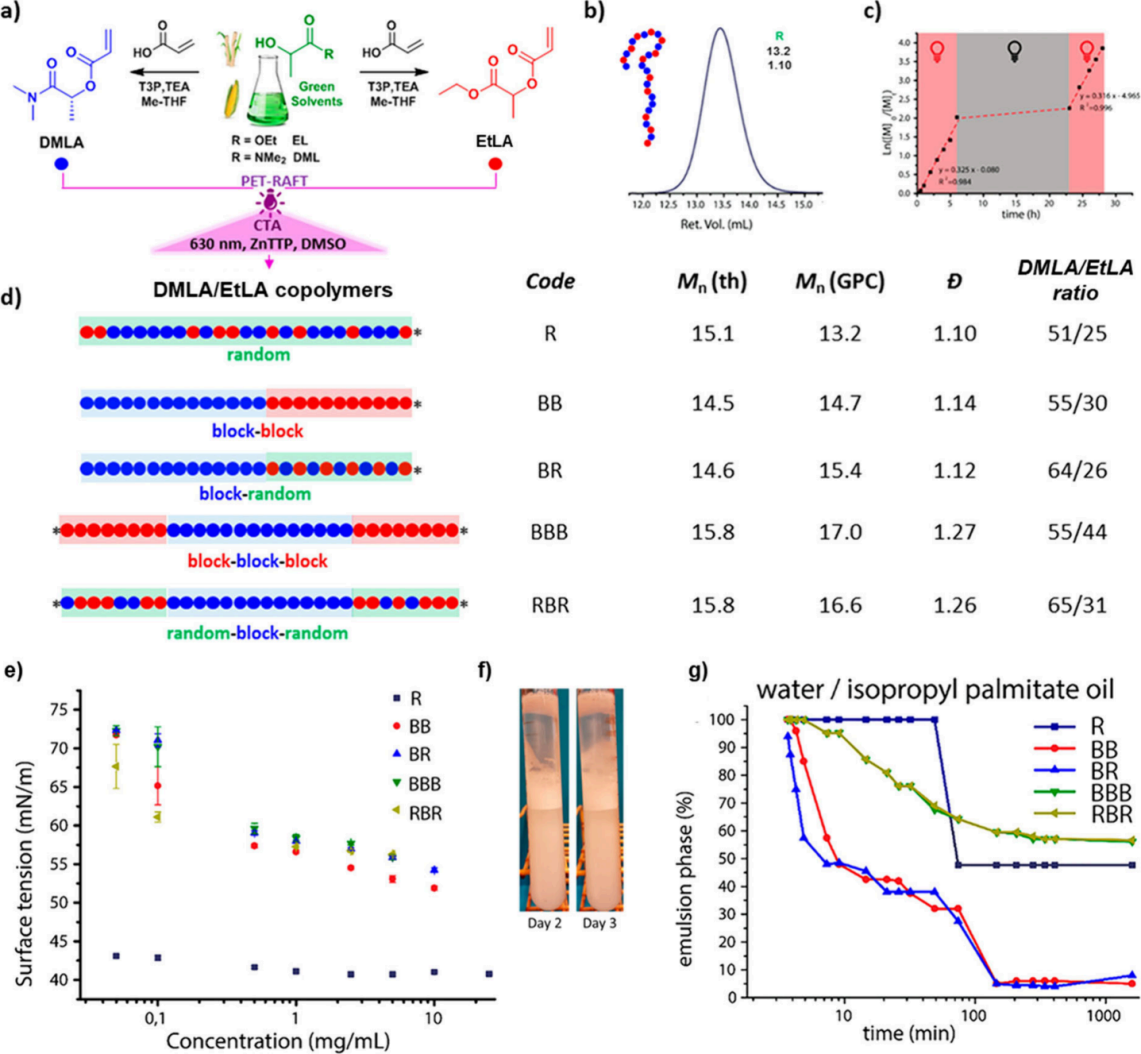
Synthesis of amphiphilic block copolymers from DMLA and EtLA and their applications as stabilizers for oil emulsions. a) Synthesis of DMLA and EtLA acrylic monomers from the corresponding green solvents. b) SEC trace of poly(DMLA-*r*-EtLA) random copolymer R. Numbers shown together with the SEC trace correspond to *M*_*n*_ (SEC, kg·mol^–1^) and *Đ*. c) Evolution of the ln([*M*]_0_/[*M*]) vs time for the ON–OFF–ON PET-RAFT random copolymerization of DMLA. d) Schematic representation of the targeted composition profiles of DMLA/EtLA copolymers together with the corresponding code, molar mass, and composition data for the synthesized copolymers. e) Comparison of surface tension as a function of the polymer concentration for the different polymer architectures. f) Digital images of the water/paraffin oil emulsions stabilized by copolymer R at different times. g) Comparison of the kinetic study of the emulsion stability over time of the 50% v/v water/isopropyl palmitate oil stabilized by 0.25% w/v of polymer. Adapted from ref (111). Copyright 2022 American Chemical Society.

Later, and inspired by the lucrative amphiphilic properties of PEtLA/PDMLA copolymers, the potential of these copolymers as surfactants for water/oil (w/o) emulsions was explored in a collaborative work lead by Raffa laboratory.^111^ In this study, a series of amphiphilic PEtLA/PDMLA copolymers with different compositions ranging from poly(DMLA-*r*-EtLA) random copolymer (R) to a poly[(DMLA-*r*-EtLA)-*b*-DMLA-*b*-(DMLA-*r*-EtLA)] triblock copolymer (RBR) with outer random segments were prepared via photoinduced electron/energy transfer-reversible addition–fragmentation chain transfer (PET-RAFT) polymerization (Figure 14a,d). This polymerization technique allowed the preparation of well-defined copolymers in all the cases, and with the possibility to control polymer chain growth, in a spatiotemporally fashion by ON–OFF light switching (Figure 14b,c). The potential of PEtLA/PDMLA copolymers as emulsion stabilizers was evaluated through surface tension measurements and emulsion stabilization of different oils in water. Comparison of surface tension as a function of copolymer concentration showed no significant difference between copolymers having block or triblock architecture (Figure 14e). Remarkably, copolymer R was found to be more surface active, displaying a decrease in surface tension of up to 42 mN·m^–1^ even at low polymer concentrations. This was attributed to the random structure allowing for dynamic adsorption of the polymer at the surface/interface, while block copolymers are known to exist in solution as “frozen” aggregates.^112^ The applicability of the copolymers as emulsion stabilizers was investigated using three different oils with varying polarities (paraffin oil, isopropyl palmitate, and sunflower oil). Regardless of the copolymer and oil employed, stable water/oil emulsions could be obtained (Figure 14f). Interestingly, it was found that the triblock copolymers proved to be the most efficient stabilizers, with a stabilization capacity of up to 40% (v/v) of water in the oil phase after several hours (Figure 14g). However, no clear trends could be identified regarding copolymer architecture and oil nature. For instance, while the random copolymer was found to be less effective in stabilizing paraffin and sunflower oil, it was found to be among the best in stabilizing isopropyl palmitate oil. This suggests that not only a single factor governs emulsion stabilization, but also a combination of factors such as hydrogen bonding or hydrophobic/hydrophilic balance plays a crucial role. Overall, these results demonstrate the promising applicability of PEtLA/PDMLA amphiphilic copolymers as surfactants for oil emulsions. However, they also point out the challenge of correlating oil polarity, polymer architecture, and emulsion stability. This lack of clear correlation would necessitate trial-and-error assays to find the optimal surfactant for each specific oil, thus hindering their industrial application. Therefore, future research should focus on studying the effects of hydrophobic/hydrophilic balance, evaluating noncovalent forces via rheology assays, and investigating polymeric architecture in detail. These efforts are crucial to establishing correlations and simplifying the design of effective surfactants for each specific type of oil.

In a similar domain, a collaborative work with Hatton laboratory demonstrated the possibility of preparing temperature-responsive nanoparticles via RAFT-mediated polymerization-induced self-assembly (PISA) of DMLA and EtLA in water.^98^ This approach involves the RAFT polymerization of DMLA in an aqueous solution to generate a hydrophilic PDMLA macromolecular chain transfer agent (macro-CTA), which was extended with EtLA in water to form amphiphilic PDMLA-*b*-PEtLA diblock copolymer nanoparticles by RAFT aqueous emulsion polymerization. When the diblock copolymer nanoparticles were analyzed by DLS, particle size diameters ranging about 74 nm were observed at temperatures ranging from 5 to 70 °C. Above these temperatures, and in the case of diblock copolymers with a considerable amount of PDMLA (DP > 50), an increase in particle size (250 nm) was observed, which was associated with the lower critical solution temperature (LCST) transition, thus confirming the thermoresponsive behavior of the system. These results support the potential use of EtLA and DMLA in PISA polymerization techniques, which are of great importance for accessing complex polymer-based biohybrid nanostructures.^113^

## 6 Well-Defined Random Copolymers from DMLA and THFLA

The application of polymeric materials in advanced applications often not only requires a precise control over the polymeric structure, but also the presence of functional groups that offer the possibility to interact with matter (e.g., biological surfaces) or sense the application of external stimuli (e.g., light, temperature) and translate it into an observable response based on physiochemical changes.^114,115^

**Figure 15:**
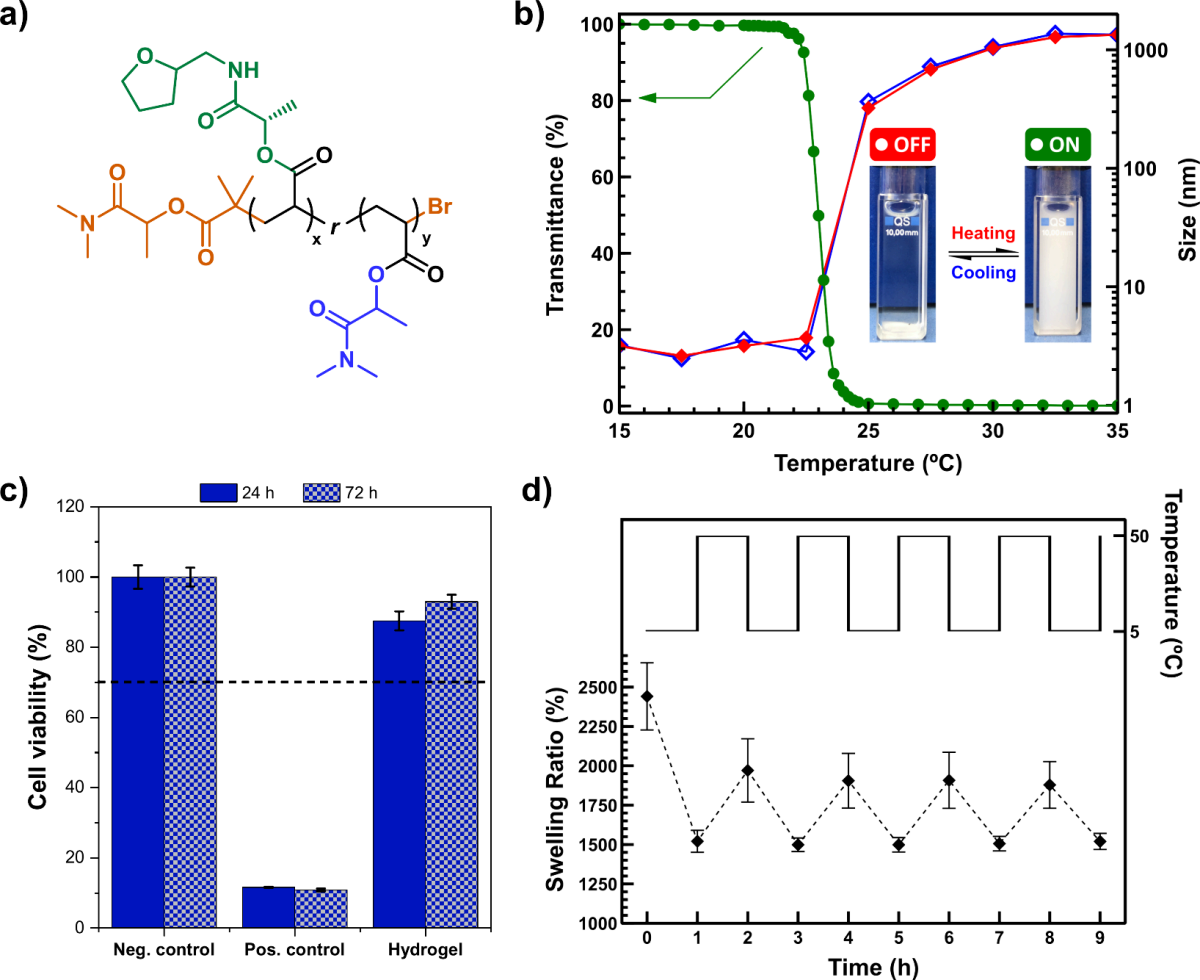
Synthesis of tunable LCST-type of acrylic (co)polymers from DMLA and THFLA and their application in the preparation of temperature-responsive hydrogels. a) Structure of poly(THFLA-*r*-DMLA). b) Transmitted laser light intensity (measured by UV/vis) and the hydrodynamic diameter measured by DLS vs temperature for PTHFLA (DP = 20, c = 5 mg·mL^–1^) (close and open symbols were obtained upon heating and cooling, respectively). The inset shows the representative digital images of PTHFLA with DP = 160 at 5 °C (left) and 25 °C (right). The ON/OFF label refers to polymer chains in a globule/flexible coil state. c) Cytotoxicity studies. d) Reversible changes between the swelling (5 °C) and deswelling (50 °C) states for poly(THFLA-*r*-DMLA) hydrogels containing 50 mol % DMLA. Adapted from ref (65). Available under a CC-BY 3.0 DEED license. Copyright 2022 Royal Society of Chemistry.

In this regard, stimuli-responsive water-soluble polymers with a lower critical solution temperature (LCST), which is the lower boundary for demixing via coil–globule transition mechanism, are highly valuable for a number of specialized applications.^116^ In these polymeric materials below the LCST, the polymer chains remain as random coils, primarily stabilized by hydrogen bonding interactions between the polymers and water, resulting in a single homogeneous phase (water-soluble). However, above the LCST, the polymeric chains collapse into globular structures, leading to phase separation (water insoluble). This temperature-induced transition is of great interest for the loading and delivering of active compounds. Therefore, the possibility of fine-tuning the LCST represents an attractive strategy to match specific applications. In this sense, one of the most popular polymers with this remarkable behavior is poly(*N*-isopropylamide) (PNIPAM) that displays a LCST around 35 °C.^117^ Owing the structural resemblance of PDMLA and its excellent solubility in water we were intrigued to see if it would exhibit similar temperature-responsive behavior. However, PDMLA exhibits a significantly higher LCST around 90 °C when the degree of polymerization is equal or higher than 50, which essentially prevent its range of application.^98^ To overcome this main limitation, we decided to evaluate the possibility to perform the copolymerization of PDMLA with other acrylic monomers with different hydrophilicity,^65^ which is one of the most well-known strategies to prepare thermoresponsive polymers with a tunable LCST.^118,119^ In this context, THFLA was selected as the comonomer for preparing the corresponding random copolymers (Figure 15a). THFLA was synthesized through a two-step process: first, the aminolysis of ethyl lactate (EL) with furfural-derived tetrahydrofurfuryl amine to produce the corresponding primary lactamide, followed by the acrylation of the secondary hydroxyl group with acrylic acid. THFLA was found soluble in water at low temperature, thus its homopolymerization was perfomed via aqueous SET-LRP mediated by nascent Cu(0) and Cu(II)Br_2_ produced “*in situ*” by the predisproportionation of Cu(I)Br in water using a DML-based initiator as mentioned in Section 4 (Figure 9a,b). To our delight, aqueous solutions of PTHFLA were clear at low temperatures (5 °C) but became cloudy at room temperature (Figure 15b, inset), confirming a temperature-responsive behavior and a foreseeable lower LCST in contrast to PDMLA owing to its higher hydrophobicity. The thermoresponsive behavior of PTHFLA was evaluated using optical transmission and variable temperature dynamic light scattering (DLS) measurements (Figure 15b). These analyses identified the temperature (23 °C) at which the transmittance of the PTHFLA solution decreased by 50%, known as the cloud point (*T*_cp_), which is associated with the LCST. This conclusion was supported by a significant increase in the hydrodynamic diameter as the temperature increase from 15 to 35 °C (Figure 15b). This transition was found reversible upon cooling suggesting that the polymer chains have the ability to change from an agglomerated and insoluble (ON) state to hydrated (OFF) and soluble state. Next, we moved to our main ambition that was develop tunable LCST-type of acrylic (co)polymers derived from LAAs. Thus, in order to manipulate the hydrophobic–hydrophilic balance, copolymerization of DMLA and THFLA was performed ranging the monomer content THFLA:DMLA from 80:20 to 30:70. Analysis of aqueous solutions of poly(THFLA-*r*-DMLA) through optical transmission measurements clearly demonstrated that the *T*_cp_ increased linearly from approximately 25 °C to around 63 °C with increasing DMLA content. This rise in *T*_cp_ is attributed to the enhanced hydrophilicity of the copolymers as the proportion of PDMLA segments increases. These results indicate the potential of these copolymers to precise tune the LCST for specific applications such as living body temperature (37 °C) or virus inactivation treatment (60 °C). Importantly, we could also cover this *T*_cp_ window by simply adjusting the molar mass of the copolymers (DP from 160 to 20) using a simple copolymer composition (51 mol % of DMLA) and the presence of salts in the aqueous solutions. Finally, these results motivated us to prepare temperature-responsive hydrogels through the free radical polymerization of THFLA and DMLA, with a specific composition to achieve a *T*_cp_ in the range of 30–40 °C, using *N*,*N*′-methylene bis(acrylamide) (MBA) as a cross-linker. The resulting hydrogel exhibited excellent biocompatibility with human dermal fibroblasts and demonstrated remarkable thermoresponsive behavior (Figure 15c,d). This behavior allowed for reversible changes in the swelling ratio and diameter due to the repeated release and uptake of water (Figure 15e), indicating that DMLA and THFLA have the potential to be used in the development of biobased soft smart materials.

## 7 Well-Defined Polymer Brushes from DMLA

**Figure 16:**
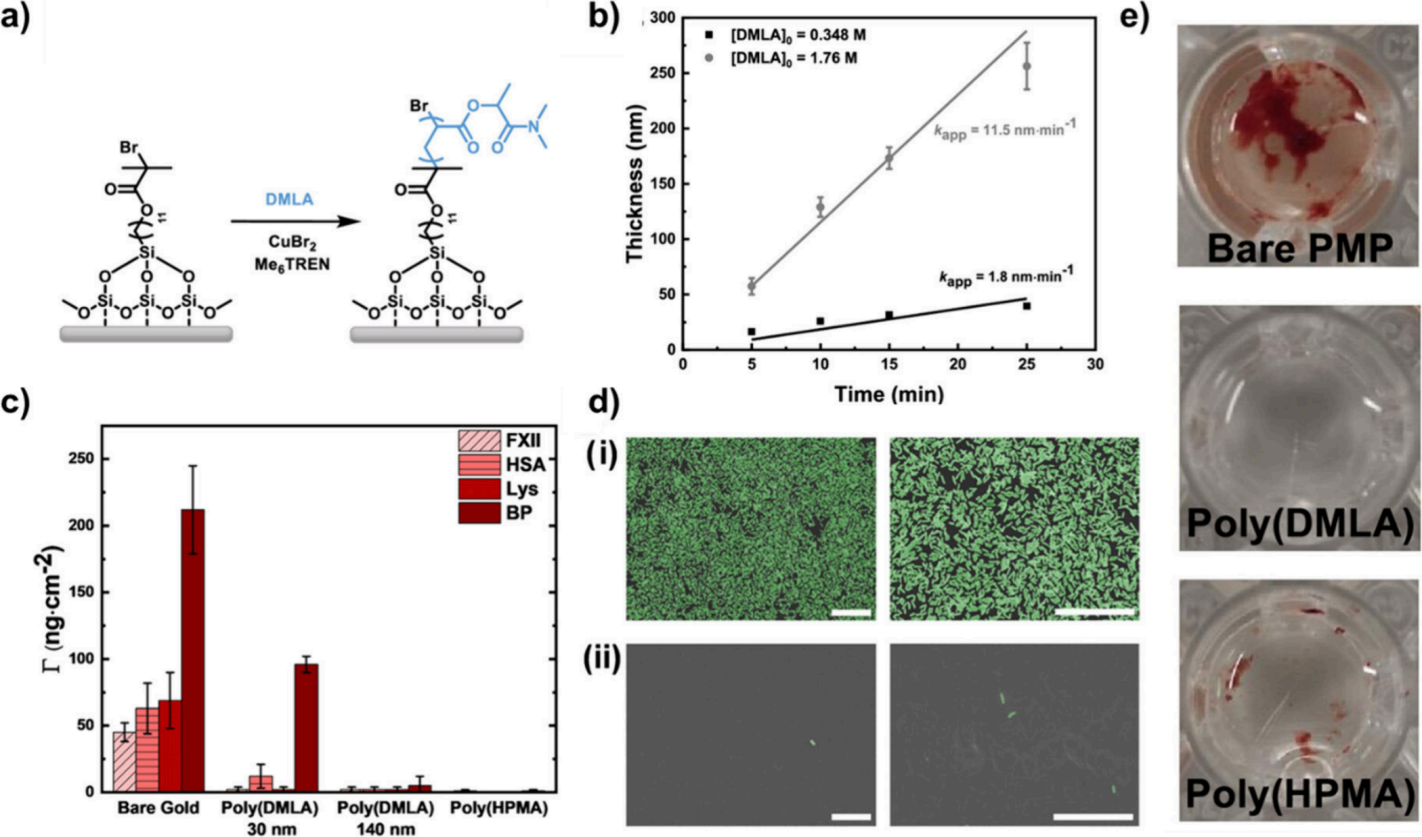
Synthesis of PDMLA brushes coatings and evaluation of its antifouling properties and hemocompatibility. a) Scheme of PDMLA brush synthesis from silicon substrates. b) Kinetics of the photoinduced SET-LRP of PDMLA brushes in DMSO (*n* = 3). c) Fouling of FXII (30 μg·mL^–1^ in PBS), HSA (5 mg·mL^–1^ in PBS), Lys (1 mg·mL^–1^ in PBS), and undiluted human BP coated and uncoated gold sensors slides (*n* = 3) d) FESEM images of (i) bare PE and (ii) PE coated with PDMLA brushes with a thickness of 140 nm after 24 h of exposure to *E. coli*. The images were false colored to enhance visualization (scale bars = 20 μm). e) Macroscopic images of the substrates after static blood contact with visible clot formation on bare PMP. Adapted from ref (120). Available under a CC-BY 3.0 DEED license. Copyright 2022 Royal Society of Chemistry.

Inebriated by the excellent hydrophilicity and biocompatibility that hydrogels composed by DMLA and THFLA exhibited, a following collaborative work led by the Rodriguez-Emmenegger laboratory explored the potential of polymer brushes based on PDMLA.^120^ In this sense, the PDMLA brushes were prepared by Cu(II)-catalyzed photoinduced radical polymerization in different substrates such as silicon, gold, poly(ethylene) (PE), and poly(4-methyl-1-pentene) (PMP) functionalized with a self-assembled monolayer (SAM) of 11-(trichlorosilyl)undecyl 2-bromo-2-methylpropanoate (Figure 16a). Silicon and gold surfaces were selected to suit specific analytical techniques as is the case of ellipsometry and fouling measurements with surface plasmon resonance (SPR), respectively, while PE its widely use in medical devices susceptible to bacterial infections, and PMP commonly employed in extracorporeal membrane oxygenators, which direct interface with blood. The kinetics of the polymerization process were evaluated by following the increase of the dry thickness over time, which turns in a linear growth of the brushes for both low (0.348 M) and high (1.76 M) concentrations of DMLA hinting that the polymerizations proceeds controlled and giving access to PDMLA brushes coatings of 30 and 140 nm (Figure 16b). When evaluating the antifouling properties of PDMLA, the initial results focused on the nonspecific adsorption of coagulation factor XII (FXII), which is responsible for initiating the coagulation process upon contact with the system. The adsorption of FXII on bare gold was found to be Γ_FXII_ = 45 ng·cm^–2^, which is sufficient to promote the initiation of the coagulation process. In contrast, PDMLA brushes completely prevented the fouling of FXII, indicating the potential of PDMLA brushes to prevent the activation of coagulation events (Figure 16c). The interaction of the PDMLA brush coatings was also evaluated by exposing them to solutions of two charged single proteins. For both proteins—human serum albumin (HSA, negatively charged) and lysozyme (Lys, positively charged)—rapid adsorption on bare gold was detected, while thinner PDMLA brushes were capable of considerably reducing fouling (by 81% in the case of HSA), while the thicker PDMLA brushes were able to prevent fouling altogether (Figure 16c). Impressed by these results, the challenging undiluted blood plasma (BP) owing its rich protein and biomacromolecule content was also evaluated. In this case, thinner PDMLA brushes reduced the fouling by 55%, and remarkably PDMLA brushed with a thickness of 140 nm display a reduction of 98% in the adsorption, which turns in negligible fouling (Figure 16c). Here, it is important to note that PDMLA brush coatings stand the comparison to the best fossil-derived brushes coating based on *N*-hydroxypropyl methacrylamide (HPMA). PHPMA brush coatings have raised some concerns due to that they are able to induce undesired immune responses and inflammatory reactions.^121^ Therefore, the excellent repelling properties shown by PDMLA brushes have the potential to replace such coatings in a near future. In addition, the antibacterial properties of PDMLA brushes were also evaluated by exposing PE and PE coated with PDMLA to *E. coli* for 24 h. After the exposition FESEM images of bare PE revealed a uniform surface covered by the bacteria, while in the coated PE only few of them could be observed, demonstrating that PDMLA brushes prevent the adhesion and colonization of bacteria (Figure 16d). After observing the remarkable antifouling performance, the compatibility with human blood and the capacity to avoid surface-induced activation of coagulation were also studied. In this case, PMP was selected as the substrate. PMP and PMP coated with PDMLA brushes were incubated with heparinized human blood. After 2 h of incubation, a macroscopic clot was observed on the surface of bare PMP, while no macroscopic thrombus could be seen on the PDMLA-coated sample. In contrast, PHMPA showed minimal traces of thrombus (Figure 16e). Overall, these findings, along with the excellent antifouling properties, suggest that PDMLA coatings could soon replace fossil-derived coatings in medical device applications.

## 8 Summary and Outlook

In this review, we have summarized the different synthetic approaches for the preparation of (meth)acrylic monomers derived from LEs and LAs and their subsequent transformation into well-defined homo- and copolymers via reversible-deactivation radical polymerization (RDRP) methods. A careful analysis of the literature supports that (meth)acrylic polymers derived from LEs and LAs hold great potential in many applications, such as stabilization of emulsions, biomedical coatings, and digital 3D printing. However, despite the exciting progress achieved, challenges and opportunities still exist. The most recognized applications of (meth)acrylic monomers derived from LEs and LAs rely on ethyl lactate acrylate (EtLA) and *N*,*N*-dimethyl lactamide acrylate (DMLA), while others remain less explored, despite having interesting properties such as tetrahydrofurfuryl lactamide acrylate (THFLA) in the preparation of smart hydrogels, or methyl- and butyl lactate acrylate in 3D printing. Therefore, future work should focus on the design of libraries of (meth)acrylic monomers derived from LEs and LAs with different properties (e.g., solubility, bulkiness). In this sense, the direct aminolysis of ethyl lactate (EL) or chemical upcycling of PLA residues with different amines or alcohols appears to be an excellent approach to introduce new functionalities into the monomeric structure. Linked to that, and as discussed in Section 2, the most effective way to install the acrylate functionality is by the direct reaction of LEs or LAs with acryloyl chloride. However, in this case, “effective” is not synonymous with “sustainable” due to the considerable amount of solvents used and the requirement of a final purification step (vacuum distillation or flash chromatography). In this sense, it is worth mentioning that future work could benefit from exploring the use of biocatalysts to conduct this transformation. For instance, chemo-enzymatic approaches using *Candida antarctica* type B lipase have been reported for the acrylation of biomass-derived precursors without the need of extra purification steps.^122^ Nevertheless, evaluation of the compatibility of esters and amides with the enzyme’s active site should be considered.

In parallel, it is important to note that, since some of the presented materials can function at the interface between material science and biology, evaluating their biodegradability, toxicity, and recyclability still requires more understanding and effort. For instance, degradation of PDMLA brush coatings presented in Section 7 could be challenging, involving a basic treatment that will only partially degrade the material via hydrolysis of ester linkages, while the carbon backbone will remain unchanged. Therefore, future work should assess the possibility of introducing labile groups between the carbon backbone to improve degradability under less demanding conditions (e.g., physiological conditions), as demonstrated with the copolymerization of fossil-derived acrylic polymers in the presence of cyclic ketene acetals.^123^ In addition, the degradability of ester linkages would benefit from being explored in neutral environments and with the use of enzymes to avoid harsh reaction conditions (e.g., basic conditions) that could affect other functionalities added in the design of functional copolymers, such as the presence of acidic groups. Therefore, it is clear that further research is necessary to elucidate the end-of-life and environmental impacts of derived (meth)acrylic polymers in LEs and LAs in various emerging applications. In this context, the depolymerization of (meth)acrylic polymers has recently emerged as a promising chemical recycling method for a circular economy, particularly through controlled depolymerization approaches that enable a gradual decrease in molecular weight.^124,125^ However, these methods have so far been applied only to commercially available and petroleum-sourced (meth)acrylic monomers. Consequently, the impact of these approaches on biobased (meth)acrylic polymers remains unexplored.

In addition, precisely engineered PLEAs and PLAAs may be important candidates for applications in chiral recognition and enantioselective catalysis, since both are chiral synthons. Consequently, building on the excellent antifouling properties shown by PDMLA brush coatings (Section 7), future works could benefit from exploring the correlation of chirality with antibacterial properties to develop the next generation of polymers derived from LEs and LAs with improved properties for specific applications. Ultimately, we anticipate that future opportunities in this field will encompass the synthesis of more complex polymeric architectures, the scaling up of both monomer synthesis and polymerization processes, and the development of poly(meth)acrylates derived from LEs and LAs for currently unexplored applications. Last but not least, we hope that this review will inspire researchers to develop new methodologies aimed at maximizing and unlocking the potential of (meth)acrylic polymers derived from LEs and LAs across disciplines, including chemical biology and materials science.
